# Focal nodular hyperplasia that mimicked a liver metastasis from a soft tissue sarcoma: a case report

**DOI:** 10.1186/s40792-017-0332-0

**Published:** 2017-04-28

**Authors:** Masataka Amisaki, Soichiro Honjo, Noriyuki Iida, Satoshi Kuwamoto, Yoshiyuki Fujiwara

**Affiliations:** 1 Division of Surgical Oncology, Department of Surgery, School of Medicine, Tottori University Faculty of Medicine, Yonago, 683-8504 Japan; 2 Division of Molecular Pathology, Department of Pathology, Tottori University Hospital, Yonago, Japan

**Keywords:** Focal nodular hyperplasia, Soft tissue sarcoma, Spindle cell sarcoma, Hepatic resection

## Abstract

**Background:**

Imaging modalities (computed tomography (CT), ultrasonography, and magnetic resonance imaging (MRI)) have only limited ability to distinguish liver focal nodular hyperplasia (FNH) from metastatic liver tumors. Here, we report a patient who underwent surgery for benign FNH that mimicked a liver metastasis from soft tissue sarcoma (STS).

**Case presentation:**

A 23-year-old man with a history of several surgeries for metastatic abdominal STS, developed a hepatic tumor accompanying peritoneal STS recurrence. He was diagnosed with a metastatic liver tumor from the STS, based on imaging studies for the hepatic tumor that showed a growing hypervascular lesion and hypo-intensity in hepatic phase on dynamic CT and MRI. However, when the liver and peritoneal tumors were resected, histological diagnosis showed the hepatic tumor to be benign liver FNH.

**Conclusions:**

Although FNH should be considered as a differential diagnosis for hypervascular hepatic tumors, it has few typical findings, and its appropriate management is controversial. A lesion strongly suspected of being a metastatic liver tumor might require surgical resection.

## Background

Soft tissue sarcomas (STS) comprise a heterogeneous group of rare solid tumors. Although only a resection is needed for cure, intra-abdominal STS frequently recurs in the liver and peritoneum even after curative resection [1, 2]. For recurrent STS, the National Comprehensive Cancer Network guideline recommends surgery if the disease is resectable [3].

In contrast, surgery is not indicated for liver focal nodular hyperplasia (FNH) because it is a common and benign focal liver lesion [4, 5], and its natural history is typically uneventful [6]. However, several clinical cases have been surgically resected due to inaccurate diagnosis [7–9], mainly because differential diagnosis of FNH includes many kinds of hypervascular hepatic tumors. When a patient has other malignant disease, diagnosis of FNH can be even more complicated.

Here, we describe a patient with peritoneal recurrence of spindle cell sarcoma (SCS)—an unclassified STS—and FNH that was misdiagnosed as an STS metastasis to the liver.

## Case presentation

A 23-year-old man had a history of two resections of SCS as the following clinical course.

**Fig. 1:**
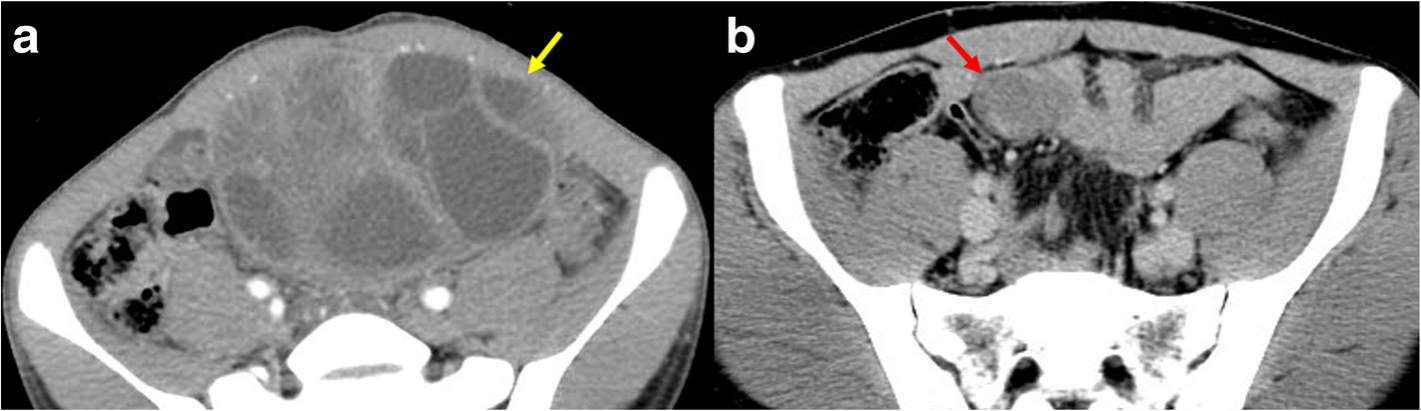
CT images for patient’s history of spindle cell sarcoma (SCS), originating from lower abdominal wall and 1st peritoneal recurrence after surgery. **a** Very large SCS stands out from lower abdominal wall (*yellow arrow*) when the patient was 13 years old. **b** Peritoneal recurrence of SCS on the mesentery over the small intestine (*red arrow*) when the patient was 22 years of age. Both lesions were curatively resected

At the initial resection at the age of 13, although the tumor was curatively resected, the SCS located on posterior layer of the rectus abdominis sheath was injured with the abdominal cavity exposed (Fig. 1a). Therefore, after his initial resection, he underwent adjuvant chemotherapy with cyclophosphamide, actinomycin-D and vincristine.

**Fig. 2:**
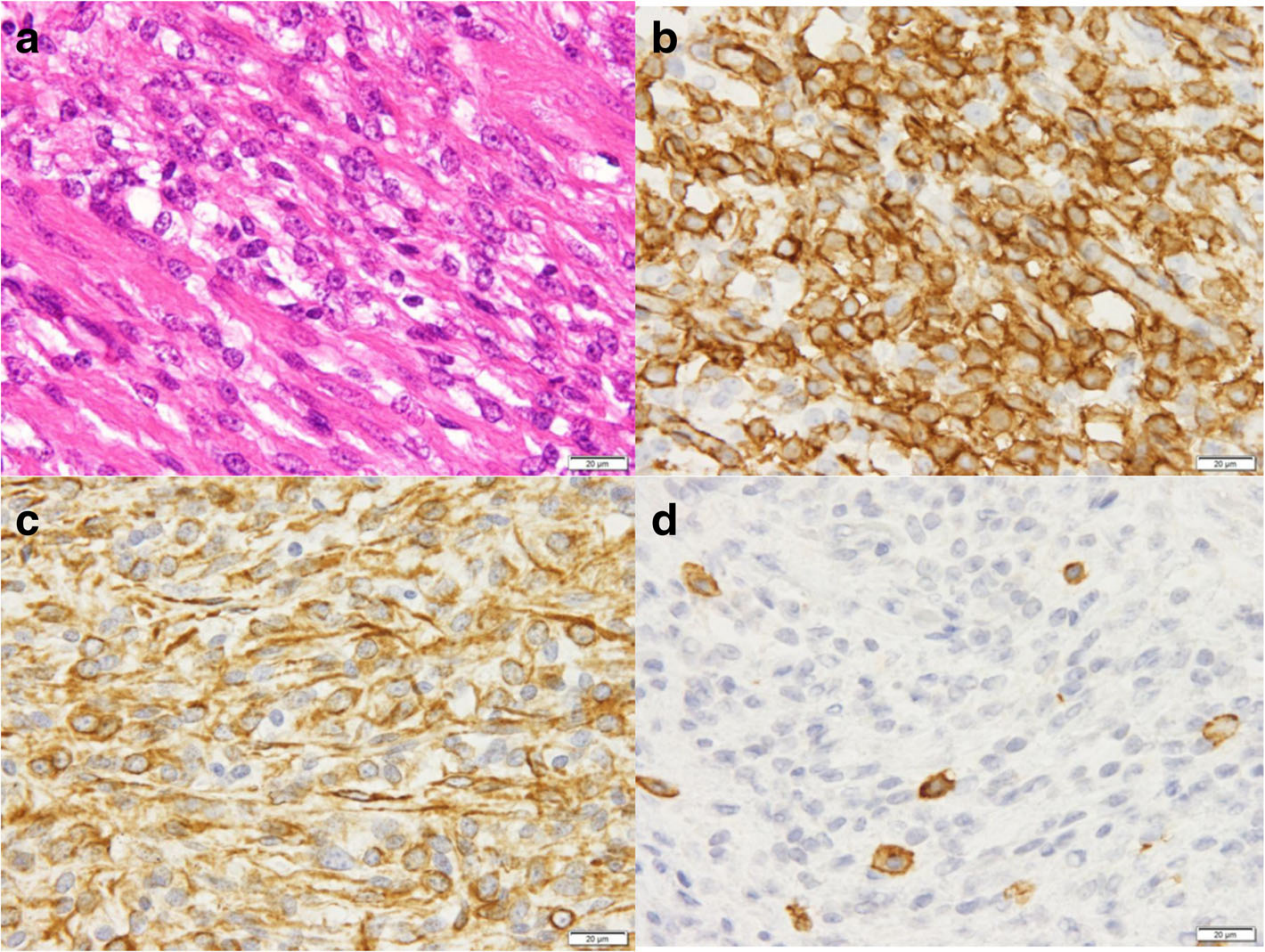
Hematoxylin-eosin (HE) staining and immunohistochemical (IHC) findings of resected spindle cell sarcoma specimen. **a** HE staining shows tumor cells with small spindle-shaped cytoplasm. **b**–**d** IHC staining shows tumor cells to be **b** CD34^+^, **c** vimentin^+^, and **d** C-Kit^−^. C-Kit^+^ cells in **d** are mast cells

At the age of 22, he was suffering from recurrence of the SCS besides the small intestine and was given curative resection (Fig. 1b). Histological examination of specimens showed an unclassified and intermediate grade SCS (Fig. 2a). Immunohistochemical findings showed the tumor cells to be positive for vimentin, TLE1, CD34, and EMA, and negative for desmin, myogenin, myoD1, SMA, CD117, S100, AE1/3, EMA, and ALK-1 (Fig. 2b). Furthermore, the fusion genes of rhabdomyosarcoma (*PAX3–FKHR* and *PAX7–FKHR*) were not detected on polymerase chain reaction-based method (data not shown).

**Fig. 3:**
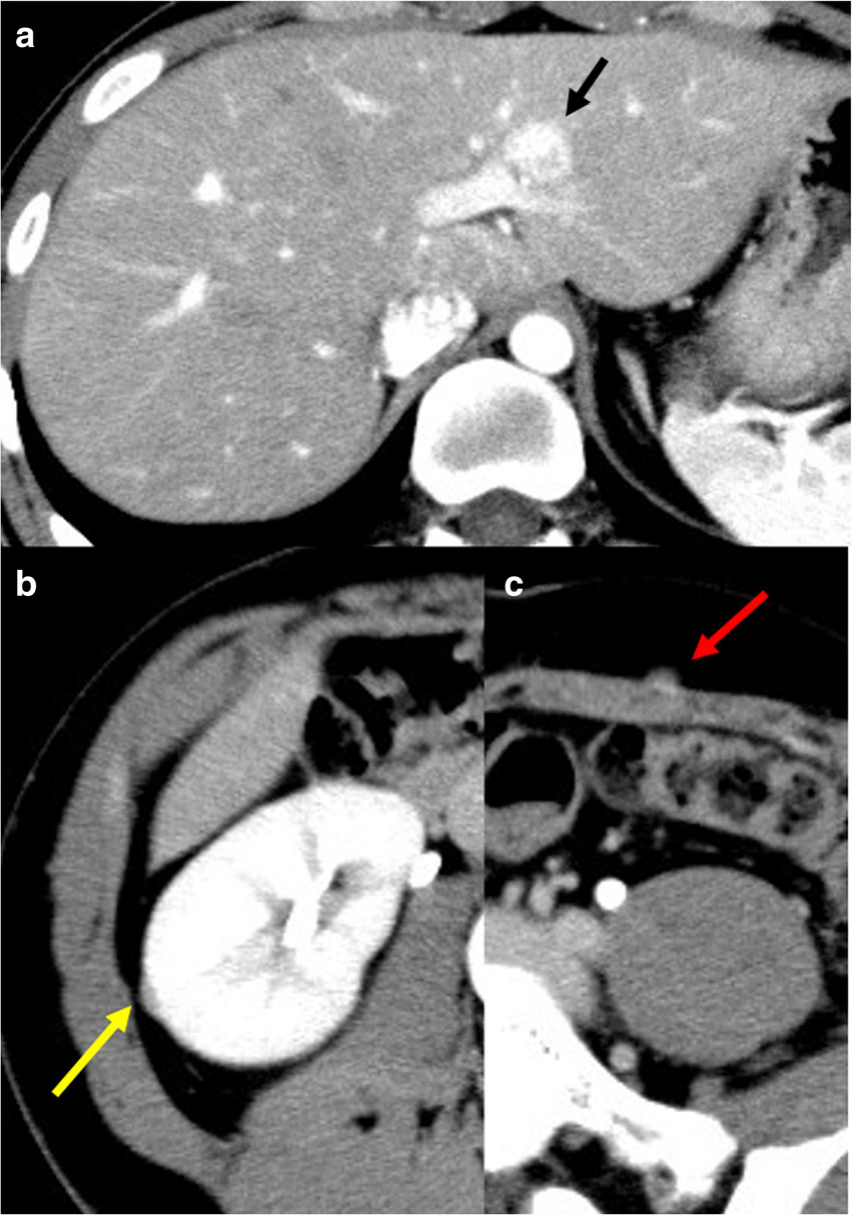
Liver tumor with intra-abdominal recurrence of spindle cell sarcoma. **a** Liver tumor is hyperdense during hepatic arterial phase (*black arrow*). **b**, **c** Two other disseminated nodules are shown beside the right kidney (**b**: *yellow arrow*) and on left anterior layer of the rectus abdominis sheath (**c**: *red arrow*)

**Fig. 4:**
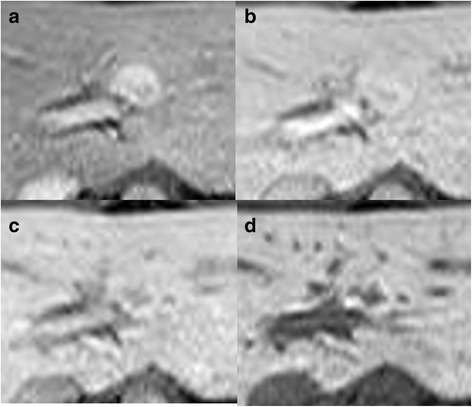
Hemodynamics of liver tumor on Gd-EOB-DPTA-enhanced MRI. Hyper-vascularity is shown on **a** arterial phase, **b** portal venous phase, and **c** delayed phase. **d** On hepatocyte phase, the hypo-intense lesion is shown with a ring-shaped hyperintense lesion

**Fig. 5:**
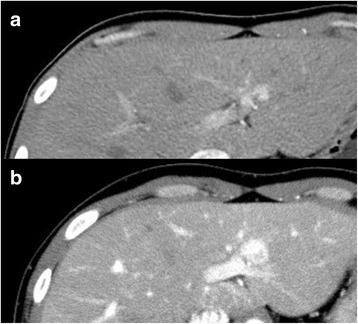
Tumor growth within 16 months. Liver lesion grew from **a** 8 mm in diameter 16 months before surgery to **b** 18 mm at surgery

**Fig. 6:**
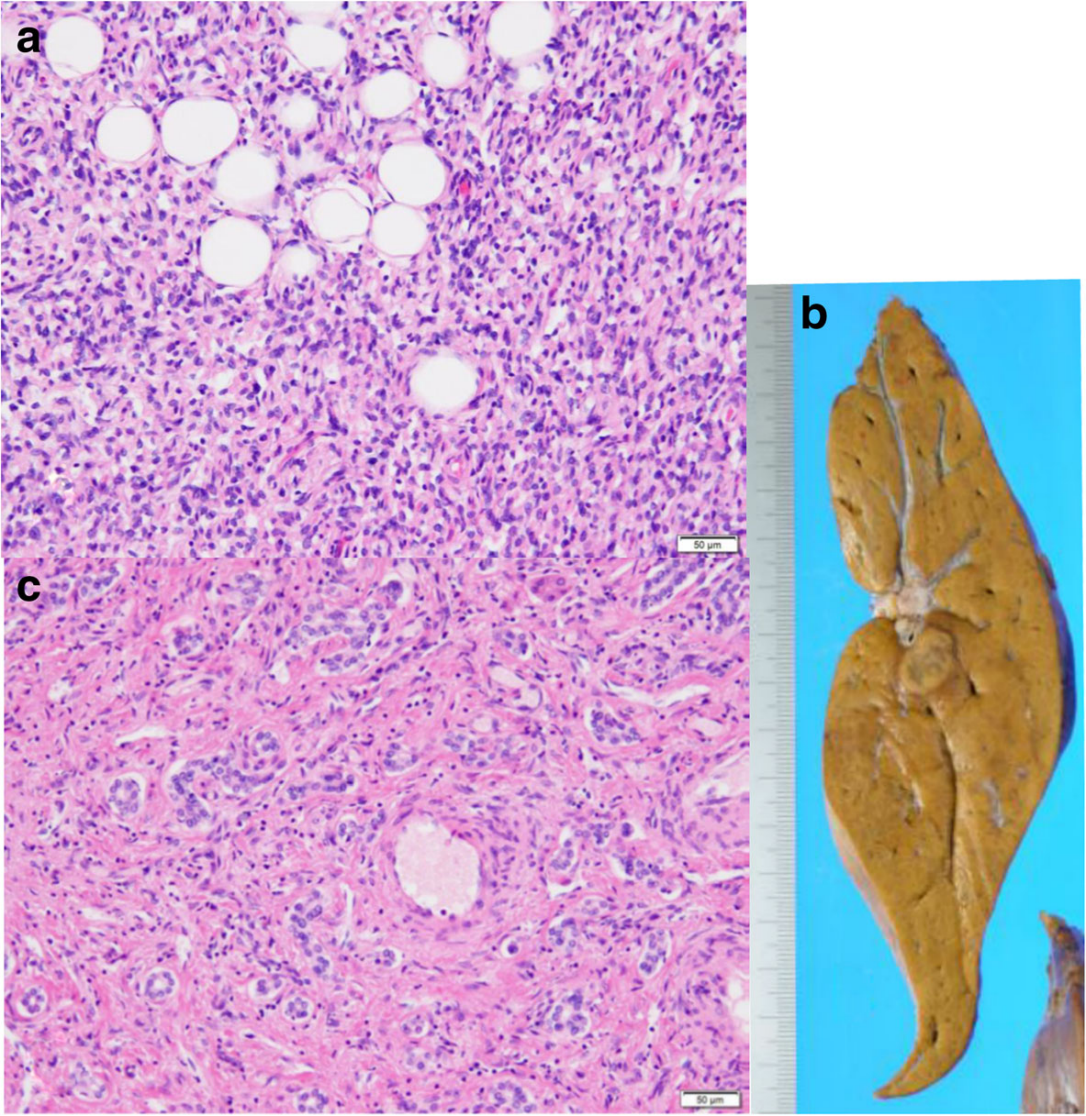
Macroscopic and microscopic findings of resected specimens. **a** Two nodules in the abdomen showed metastatic soft tissue sarcoma, with small-nuclei spindle cells that grow invasively into adipose tissue. **b**, **c** The hepatic tumor contained **b** a small radiating scar, and **c** included large portal tracts and proliferated bile ducts

During the follow-up period, a solitary tumor in the lateral segment of the liver and two other tumors that are located beside the right kidney and on left anterior layer of the rectus abdominis sheath were newly diagnosed through contrast-enhanced computed tomography (CECT; Fig. 3a (liver tumor), Fig. 3b, c (small nodules)). The Gd ethoxybenzyl diethylenetriamine pentaacetic acid (Gd-EOB-DPTA)-enhanced magnetic resonance imaging (MRI) for the hepatic tumor showed low intensity on T1-weighted image (WI) and slightly high intensity on T2WI, as a hypervascular lesion in dynamic study (Fig. 4a–c), and partly low-intensity area in the hepatocyte phase (Fig. 4d). The hepatic tumor almost doubled in diameter within 18 months (Fig. 5). Based on these results, the hepatic tumor was diagnosed as a liver metastasis of SCS. No abnormalities were observed in laboratory findings, including tumor makers (CEA, CA19-9, AFP, and PIVKA-II). Liver function was preserved, and hepatitis B surface antigen and hepatitis C antibody were both negative. Therefore, we performed a left liver lobectomy and curatively resected the two nodules in his abdomen. The operation took 391 min with 180 ml of blood loss. The postoperative course was favorable, and the patient was discharged on postoperative day 11. Histopathological examination showed recurrent SCS in the abdominal nodules (Fig. 6a); however, the hepatic tumor was diagnosed as benign FNH (Fig. 6b, c).

## Discussion

Surgical resection can provide the potential for cure in patients with recurrence of STS. The local control rate at 5 years after resection was 85% [10]. In contrast, patients with unresectable lesion have poor prognosis. Reflecting these facts, patients with advanced tumor have poor prognosis: Five-year survival rates for stages I, II, and III are 98, 81, and 56%, respectively, according to the tumor, node, and metastasis stage grouping [11]. Therefore, adequate diagnosis of the recurrence and resection for selected patients are needed for treatment of STS.

As this patient had both recurrent SCS—an unclassified STS—and liver FNH, distinguishing the FNH from a hepatic metastasis was difficult. Other literature also reported some FNHs that were diagnosed as metastases in patients with concurrent malignancies, who therefore underwent resections, including two patients with insulinomas and one with renal cell carcinoma (Table 1) [8, 12, 13]. In these types of malignancy, surgery is recommended for recurrent disease, if complete resection is possible.

**Table 1:** Reported cases of resected FNH and concurrent malignancies

Year	Authors	Age, gender	Concurrent malignancy (location)	Past history of recurrence	Diagnostic modality
2015	Jerraya H, et al.	59, F	Insulinoma (pancreas)	NA	NA
2015	Jung SY, et al.	11, F	Insulinoma (pancreas)	Non	US, CT, MRI
2009	Wheeler YY, et al.	62, M	Renal cell carcinoma (right kidney)	Non	CECT
2015	Present case	23, M	SCS (abdominal)	Abdominal	US, CECT, MRI

FNH typically shows a characteristic enhancement pattern on CT or MRI [14–16]: early nodular arterial enhancement and isodense appearance on portal venous phase [17]. Nevertheless, some FNHs show atypical imaging and are therefore difficult to diagnose accurately. The reported diagnostic ability in determining benign or malignant disease for CT scans is 78% specific [18] that of MRI is 96.6% sensitive and 87.6% specific [19]. To solve this problem, new modalities such as contrast-enhanced ultrasonography and shear-wave elastography [20] have been developed and assessed for diagnostic ability. However, about 10% of FNH are not accurately diagnosed preoperatively.

In the present case, a characteristic finding, such as a “central scar,” was not present. Also, in the MRI hepatobiliary phase, some atypical hemodynamic aberrations (such as the hypo-intense lesion with ring-like hyperintensity; Fig. 4d) suggested a metastatic liver tumor. Moreover, tumor growth during the observation period also suggested a malignant tumor, as FNHs rarely grow [21]. Mathieu et al. reported that tumor growth was observed in only 1.9% of FNH [6]. Thus, atypical imaging and tumor-like characteristics made accurate preoperative diagnosis difficult in this case.

Because the guideline offers no recommendation for preoperative histological diagnosis of resectable STS [2, 3], histological confirmation was not considered because surgery was the only curative treatment for SCS, and this patient had other peritoneal tumors that were highly suspected to be metastatic tumors.

Consequently, in the present case, surgical resection for liver tumors might be unnecessary according to the guideline [22]. Retrospectively, a preoperative histological confirmation for a hepatic lesion should have been performed. However, limited sampling size obtained with an aspiration biopsy might have also led to a misdiagnosis or to underestimating the malignancy [23, 24]. Therefore, a possibly malignant liver lesion should be resected. A conclusive preoperative diagnostic method for malignancy of liver tumors should be established; otherwise, clinicians should carefully consider the indication for surgery against the possibility of malignancy.

## Conclusions

In conclusion, decisions for surgical resection should depend on details of the clinical situation, such as coexistence of malignancy or enlargement of FNH over time. However, a conclusive method for diagnosing FNH should be developed to avoid unnecessary surgery.

## Abbreviations

CECT: Contrast-enhanced computed tomography
FNH: Focal nodular hyperplasia
Gd-EOB-DPTA: Gd ethoxybenzyl diethylenetriamine pentaacetic acid
MRI: Magnetic resonance imaging
SCS: Spindle cell sarcoma
STS: Soft tissue sarcoma
